# Per- and Polyfluoroalkyl Substances and Endometriosis: A Systematic Review and Meta-Analysis

**DOI:** 10.3390/toxics14040337

**Published:** 2026-04-17

**Authors:** Sarah Pilling, Kerry Mitchell, Prakash V. A. K. Ramdass

**Affiliations:** Department of Public Health and Preventive Medicine, St. George’s University School of Medicine, St. George FZ818, Grenada; spilling@sgu.edu (S.P.); kmitche3@sgu.edu (K.M.)

**Keywords:** perfluoroalkyl substances, polyfluoroalkyl substances, PFASs, PFSAs, PFCAs, endometriosis, endometrioma, meta-analysis

## Abstract

Per- and polyfluoroalkyl substances (PFASs) are persistent endocrine-disrupting chemicals implicated in reproductive dysfunction. Epidemiologic evidence examining their association with endometriosis remains inconsistent. Thus, we conducted a PRISMA-compliant systematic review and meta-analysis using PubMed, Embase, EBSCO Host, and Google Scholar databases. RStudio software was used for all analyses. Random-effects or fixed-effects model was applied to estimate pooled odds ratios (ORs) and standardized mean difference (SMD) in PFAS levels between endometriosis patients and controls. Heterogeneity was assessed using I^2^ statistics. Publication bias was evaluated using funnel plots, and Egger’s and Begg’s tests. Twelve studies met the inclusion criteria for the systematic review and eleven were included in the quantitative synthesis. Overall, PFSAs (OR: 1.50; 95% CI: 1.12–2.00) and PFCAs (OR: 1.46; 95% CI: 1.12–1.90) were significantly associated with increased odds of endometriosis, particularly PFOS and PFOA. However, analyses of pooled SMD did not demonstrate consistent concentration differences between endometriosis cases and controls. Heterogeneity was moderate to high for most compounds. Funnel plot symmetry and Egger’s and Begg’s tests suggest no publication bias. Exposure to PFASs, particularly PFOS and PFOA, may be associated with increased odds of endometriosis. Further prospective studies incorporating mixture modeling and emerging PFASs are warranted.

## 1. Introduction

Endometriosis is a disease in which tissue similar to the lining of the uterus (endometrium) is present outside the normal uterine cavity, and this misplaced tissue is typically accompanied by an inflammatory response [1]. It is clinically characterized by a range of debilitating symptoms, most commonly chronic pelvic pain, severe dysmenorrhea, deep dyspareunia, and infertility, which together can profoundly impact physical, mental, and social well-being [2]. While worldwide data show that endometriosis affects approximately 5% of women in the general population and 10% of women of reproductive age, its prevalence rises dramatically in specific clinical populations, affecting up to 42% of those with gynecological symptoms and 38% of women with infertility [3,4].

Although the exact pathogenesis of endometriosis is multifactorial and not fully understood, it is thought to involve complex interactions among genetic predisposition [5,6], hormonal influences [7], and immunological factors [8]. One of the most widely accepted explanations is Sampson’s theory of retrograde menstruation, which proposes that endometrial cells flow backward through the fallopian tubes during menstruation and enter the peritoneal cavity) [9]. These cells may then implant on peritoneal surfaces, form ectopic lesions, and initiate a chronic inflammatory response. Emerging evidence also suggests that environmental exposures, particularly to endocrine-disrupting chemicals such as per- and polyfluoroalkyl substances (PFASs), may contribute to disease development and progression [10].

PFASs are a large class of synthetic fluorinated compounds widely used in industrial applications and consumer products, including, but not limited to, textiles, food packaging, non-stick cookware, cosmetics, and electronics, thus posing significant and persistent exposure risks [11,12]. The PFAS compounds most commonly investigated in epidemiological studies of endometriosis include perfluorooctanesulfonic acid (PFOS), perfluorooctanoic acid (PFOA), perfluorohexanesulfonic acid (PFHxS), and perfluorononanoic acid (PFNA), which are often detected in human serum and have long biological half-lives [10]. PFAS exposure is linked to a wide range of health effects, even at low exposure scenarios, with evidence suggesting significant immune, metabolic, and reproductive toxicity. Recent studies suggest that PFASs toxicity may manifest as disrupted ovarian steroidogenesis [13], altered estrogen and progesterone signaling [14], impaired immune regulation, and an overall increased systemic inflammatory state [15]. In biological females, these mechanisms are highly relevant to the pathophysiology of endometriosis [2].

However, epidemiologic findings examining the association between PFAS exposure and endometriosis have been inconsistent [10,16,17]. These inconsistencies may be explained by differences in study design, variations in population characteristics, differences in PFAS exposure assessment methods and biological sample, as well as variability in diagnostic criteria for endometriosis. Given the widespread exposure to PFAS and the substantial morbidity associated with endometriosis, clarifying this relationship is of significant clinical and public health importance. Therefore, the aim of this study was to systematically review and quantitatively synthesize the available epidemiologic evidence on the association between PFAS concentrations and endometriosis.

## 2. Materials and Methods

### 2.1. Registration and Search Strategy

This systematic review and meta-analysis was conducted in accordance with the Preferred Reporting Items for Systematic Reviews and Meta-Analyses (PRISMA) guidelines [18]. The protocol was registered with PROSPERO in September 2024 (Registration ID: CRD42024591364). A comprehensive literature search was performed in the following electronic databases: PubMed, Embase, EBSCO Host, and Google Scholar. The search was conducted from database inception to January 2026. The search strategy employed the following key terms combined using Boolean operators: (“endometriosis” OR “endometrioma”) AND (“PFAS” OR “Per- and Poly-fluoroalkyl Substances”). The search was limited to studies published in the English language only.

### 2.2. Study Selection and Inclusion Criteria

Titles and abstracts of all retrieved records were independently screened by two reviewers (S.P. and P.R.) for relevance, followed by a full-text review of potentially eligible studies. Only original epidemiological studies relating to endometriosis and PFAS were included, comprising cross-sectional, cohort, case–control, and ecological designs. The following document types were excluded: conference proceedings, case reports, letters, editorials, opinion pieces, conference abstracts, and review articles. Additionally, animal studies and in vitro experiments were excluded as they did not meet the human-based design criteria of this review.

### 2.3. Data Extraction

The following data were extracted from each included study: first author’s last name, year of publication, country where the research was conducted, study design, sample size, criteria for endometriosis diagnosis, type of biological sample used for PFAS analysis, specific PFAS compounds measured, and relevant statistical data required to estimate odds ratios and mean differences. In studies where data were presented as medians and interquartile ranges, the mean and standard deviation were estimated using the approach described by Wan et al. [19]. Data on PFAS were extracted and categorized according to chemical class, chain length, and production history. PFAS compounds were first classified as perfluoroalkyl or polyfluoroalkyl substances, and then further grouped by functional group into sulfonic acids (perfluoroalkyl sulfonic acids [PFSAs]) and carboxylic acids (perfluoroalkyl carboxylic acids [PFCAs]) [20]. PFAS were also categorized by carbon chain length as follows: long-chain PFAS: ≥7 perfluorocarbon atoms for PFCAs and ≥6 perfluorocarbon atoms for PFSAs; short-chain PFAS: 3–6 perfluorocarbon atoms for PFCAs and 4–5 perfluorocarbon atoms for PFSAs [20]. PFAS were additionally categorized by production history as legacy (long-chain PFAS historically produced and now phased out in many developed countries), alternative (short-chain PFAS used as replacements for legacy compounds), and emerging (newer PFAS chemistries and polyfluorinated compounds that remain less well characterized) [21]. In addition to the study characteristics and PFAS exposure data, detailed information on confounding variables adjusted for in each study was extracted. These included age, body mass index (BMI), parity, smoking status, race/ethnicity, oral contraceptive use and socioeconomic factors (SES).

### 2.4. Quality Assessment

The methodological quality of the included studies was assessed using the Newcastle-Ottawa Scale (NOS) [22]. The NOS was originally developed for cohort and case–control studies; however, for cross-sectional studies, a modified version of the NOS was used, as described in previous systematic reviews [23].

For case–control and cross-sectional studies, the NOS assigns scores across three key domains: Selection (adequacy of case definition, representativeness of cases, selection of controls, and definition of controls); Comparability (comparability of cases and controls based on study design or analysis); and Exposure (ascertainment of exposure, use of the same ascertainment method for cases and controls, and non-response rate). The maximum achievable score for case–control studies is nine stars.

For cohort studies, the NOS assesses: Selection (representativeness of the exposed cohort, selection of the non-exposed cohort, ascertainment of exposure, and demonstration that the outcome of interest was absent at study initiation); Comparability (comparability of cohorts based on study design or analysis); and Outcome (assessment of outcome, adequacy of follow-up length for outcomes to occur, and completeness of follow-up). Cohort studies can also receive a maximum score of nine stars.

### 2.5. Statistical Analysis

For studies that reported PFAS concentrations in tertiles or quartiles, odds ratios were calculated using the lowest tertile or quartile as the reference category, with the highest tertile or quartile representing the exposure group. Studies that did not present raw case or control counts by exposure level were included if they provided odds ratios and corresponding 95% confidence intervals (CIs).

All statistical analyses were performed using RStudio software (version 4.4.1), Vienna Austria. Forest plots were generated to display pooled odds ratios and mean differences. Subgroup analyses were conducted based on PFAS chemical classification (perfluoroalkyl sulfonic acids [PFSAs] and perfluoroalkyl carboxylic acids [PFCAs]). Sensitivity analyses were performed to evaluate the robustness and stability of the pooled estimates, particularly in analyses where heterogeneity was substantial (I^2^ > 50%). Leave-one-out analyses were conducted by sequentially removing one study at a time to assess the influence of individual studies on the overall effect size. In addition, influence diagnostics, including externally studentized residuals, Cook’s distance, DFFITS values, covariance ratios, hat values, and study weights, were used to identify potential outliers and influential studies, and Baujat plots were generated to assess each study’s contribution to heterogeneity and overall effect size. Publication bias was assessed using funnel plots, supplemented by Begg’s and Egger’s tests [24,25]. To allow pooling of studies reporting different effect measures, odds ratios and standardized mean differences were converted to a common metric using established methods based on the logistic distribution (Chinn, 2000) [26]. Statistical significance was set at *p* < 0.05.

## 3. Results

### 3.1. Study Selection

**Figure 1:**
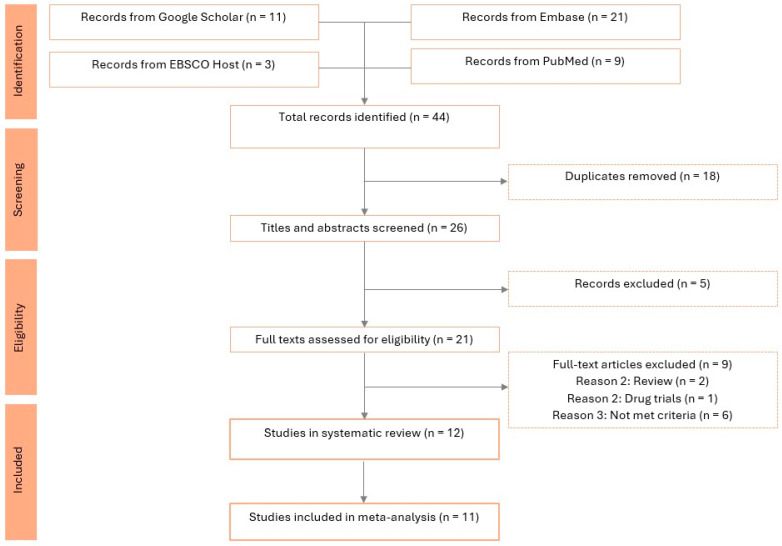
PRISMA flow diagram.

Our comprehensive literature search of the four databases yielded a total of 44 records. After the removal of duplicates, 26 unique titles and abstracts remained for screening. Following screening, 21 full-text articles were further screened for inclusion. Consequently, 12 studies met the inclusion criteria and were included in the systematic review. Among these, 11 studies provided sufficient data for quantitative synthesis and were included in the meta-analysis. One ecological study [27] was excluded from the meta-analysis because FPAS was measured as an environmental exposure in drinking water rather than in body fluids. The included studies are shown in the PRISMA flow diagram in Figure 1.

### 3.2. Study Characteristics and Quality Assessment

Table 1 provides details on the study characteristics and the quality assessment based on the NOS. A total of 12 studies [10,16,17,27,28,29,30,31,32,33,34,35] were included in this systematic review, comprising 11 studies for meta-analysis and one large ecological cohort study included only in the qualitative synthesis. The studies were conducted across eight countries, including the USA (*n* = 5), China (*n* = 2), France (*n* = 2), and one each from Australia, Spain, and Sweden. The studies were conducted from 2012 to 2025. Study designs varied and included case–control (*n* = 6), cross-sectional (*n* = 4), and ecological cohort (*n* = 1) designs, with one simulation study also included. Sample sizes ranged from 42 to 29,106 participants.

Endometriosis diagnosis was confirmed by the gold standard of laparoscopy, laparotomy, or histological verification in the majority of studies (*n* = 8). Two studies relied on self-reported physician diagnosis, one used infertility etiology as a proxy, and one utilized International Classification of Diseases (ICD) codes from a national registry [36]. PFAS exposure was measured primarily in serum (*n* = 6) and plasma (*n* = 3), with one study each utilizing follicular fluid, endometrial tissue, and drinking water contamination as the exposure matrix.

Methodological quality was assessed using the NOS. Among the case–control and cohort studies, scores ranged from 5 to 9, with five studies achieving the maximum score of 9 stars, indicating high methodological quality. Cross-sectional studies received scores ranging from 5 to 8. Overall, the included studies demonstrated moderate to high quality, with the primary sources of bias arising from self-reported outcome ascertainment and inadequate control for certain confounders in lower-scoring studies.

**Table 1:** Characteristics of included studies.

Study	Country	Study Design	Endometriosis Diagnosis	PFAS Sample	Confounders	Sample Size	NOS Score
Louis, 2012 [28]	USA	Case–control	Laparotomy/laparoscopy	Serum	Age, BMI, Parity	473	8
Campbell, 2016 [29]	USA	Cross-Sectional	Self-reported	Serum	Age, Race/Ethnicity, BMI, Smoking, SES	753	5
Ngueta, 2017 [30]	USA	Case–control	Laparotomy/laparoscopy	Serum	OCP use	201	5
Wang, 2017 [31]	China	Case–Control	Laparoscopy	Plasma	Age, BMI, Parity	335	9
Kim, 2020 [32]	Australia	Cross-Sectional	Infertility etiology factor	Follicular fluid	Age	42	5
Matta, 2022 [33]	France	Case–Control	Histological confirmation	Serum	Age, BMI	87	8
Ao, 2024 [17]	China	Case–Control	Laparoscopy	Plasma	Age, BMI	574	9
Lefebvre, 2024 [34]	France	Case–Control	Histological confirmation	Serum	Age, BMI, Parity	137	9
De Haro-Romero, 2024 [10]	Spain	Case–Control	Histological confirmation	Plasma	Age, BMI, Parity, Smoking	132	9
Marroquin, 2025 [35]	USA	Cross-Sectional	Laparotomy/laparoscopy	Endometrium	Age, Race, BMI, Smoking, Study site	434	8
Zhang, 2025 [16]	USA	Cross-Sectional	Self-reported	Serum	Age, BMI, Race/Ethnicity, Smoking, SES	1069	6
	**Systematic Review Only**						
Hammarstrand, 2021 [27]	Sweden	Ecological	ICD codes [37]	Drinking water	Age	29,106	8

PFAS—Per- and polyfluoroalkyl substances; NOS—Newcastle-Ottawa Scale; BMI—body mass index; SES—socioeconomic status; OCP—oral contraceptive pill; ICD—International Classification of Diseases.

### 3.3. PFAS Investigated in the Included Studies

Across the 12 included studies, the most frequently investigated PFAS were perfluorooctanesulfonic acid (PFOS), perfluorooctanoic acid (PFOA), Perfluorohexanesulfonic acid (PFHxS), perfluorononanoic acid (PFNA), and perfluorodecanoic acid (PFDA), with PFOS and PFOA each analyzed in all 12 studies. Less frequently studied compounds included perfluoroundecanoic acid (PFUnDA), perfluoroheptanoic acid (PFHpA), perfluorododecanoic acid (PFDoDA), and perfluoroheptanesulfonic acid (PFHpS). By chemical class [38], perfluoroalkyl sulfonic acids (PFSAs) were consistently represented, with PFOS and PFHxS being the most widely studied. Perfluoroalkyl carboxylic acids (PFCAs) were more diverse, with a broader range of chain lengths analyzed across studies. This distribution highlights a research focus on legacy long-chain PFAS, while emerging and short-chain alternatives remain comparatively understudied in the context of endometriosis. Details are shown in Table 2.

### 3.4. Systematic Review Findings

When examined collectively, the evidence suggests that variability across studies may be explained by methodological differences rather than true inconsistency in the association between PFAS exposure and endometriosis. First, studies that categorized PFAS exposure into tertiles or quartiles [10,16,17,28,29,30] were more likely to report significant associations compared with studies analyzing PFAS as continuous variables [31,33,34,35], suggesting potential threshold or non-linear exposure–response relationships. Second, studies using surgically confirmed endometriosis [10,17,28] tended to report more consistent associations than studies relying on self-reported diagnosis [33,34], likely due to reduced outcome misclassification. Third, studies that evaluated PFAS as mixtures [16,17,33,35] often reported stronger or more consistent associations compared with studies examining individual compounds [28,29,30,31,32], supporting the possibility of additive or synergistic effects from combined PFAS exposures. Together, these findings suggest that differences in exposure categorization, diagnostic accuracy, and analytical approach may explain much of the heterogeneity observed across studies.

### 3.5. Perfluoroalkyl Sulfonic Acids and Odds of Endometriosis

**Figure 2:**
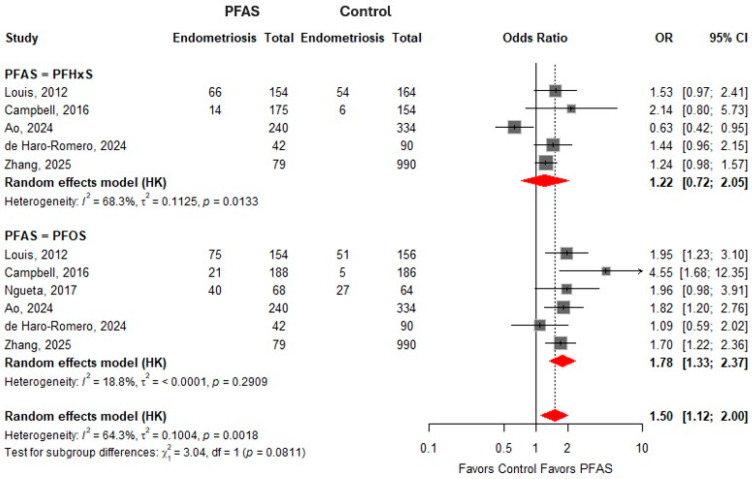
The meta-analysis of perfluoroalkyl sulfonic acids and the odds of endometriosis. PFHxS—Perfluorohexanesulfonic acid [10,16,17,28,29]; PFOS—perfluorooctanesulfonic acid [10,16,17,28,29,30].

For PFHxS, the pooled random-effects estimate suggested a positive but not statistically significant association with endometriosis (OR = 1.22, 95% CI: 0.72–2.05), with substantial heterogeneity observed across studies (I^2^ = 68.3%). Individual study estimates varied, with some studies suggesting increased odds and others showing null or inverse associations, contributing to the overall heterogeneity. For PFOS, the pooled analysis demonstrated a statistically significant association with endometriosis (OR = 1.78, 95% CI: 1.33–2.37), with low heterogeneity (I^2^ = 18.8%), indicating relatively consistent findings across studies. The overall pooled estimate for perfluoroalkyl sulfonic acids combined showed a significant association with endometriosis risk (OR = 1.50, 95% CI: 1.12–2.00), although heterogeneity was moderate (I^2^ = 64.3%). The test for subgroup differences between PFHxS and PFOS was not statistically significant (*p* = 0.0811), suggesting that while PFOS showed a clearer association, the difference between individual sulfonic acids was not statistically significant. This is shown in Figure 2.

### 3.6. Perfluoroalkyl Carboxylic Acids and Odds of Endometriosis

**Figure 3:**
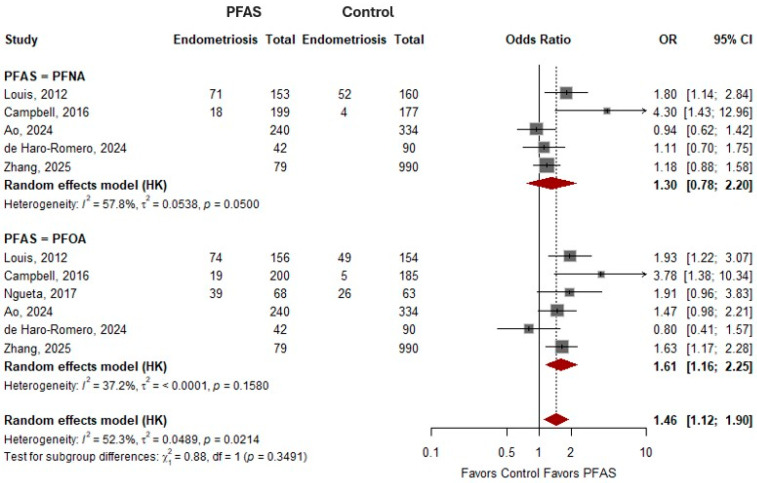
The meta-analysis of perfluoroalkyl carboxylic acids and the odds of endometriosis. PFNA—Perfluorononanoic acid [10,16,17,28,29]; PFOA—perfluorooctanoic acid [10,16,17,28,29,30].

For PFNA, the pooled random-effects estimate suggested a positive but not statistically significant association with endometriosis (OR = 1.30, 95% CI: 0.78–2.20), with moderate heterogeneity observed across studies (I^2^ = 57.8%). Individual study estimates varied, with some studies reporting increased odds while others showed null associations, contributing to the observed heterogeneity. For PFOA, the pooled analysis demonstrated a statistically significant association with endometriosis (OR = 1.61, 95% CI: 1.16–2.25), with low to moderate heterogeneity (I^2^ = 37.2%), indicating relatively consistent findings across studies. The overall pooled estimate for perfluoroalkyl carboxylic acids combined showed a statistically significant association with endometriosis risk (OR = 1.46, 95% CI: 1.12–1.90), with moderate heterogeneity (I^2^ = 52.3%). The test for subgroup differences between PFNA and PFOA was not statistically significant (*p* = 0.3491), suggesting that the associations did not significantly differ between individual carboxylic acids. This is shown in Figure 3.

### 3.7. SMD of Perfluoroalkyl Sulfonic Acids

**Figure 4:**
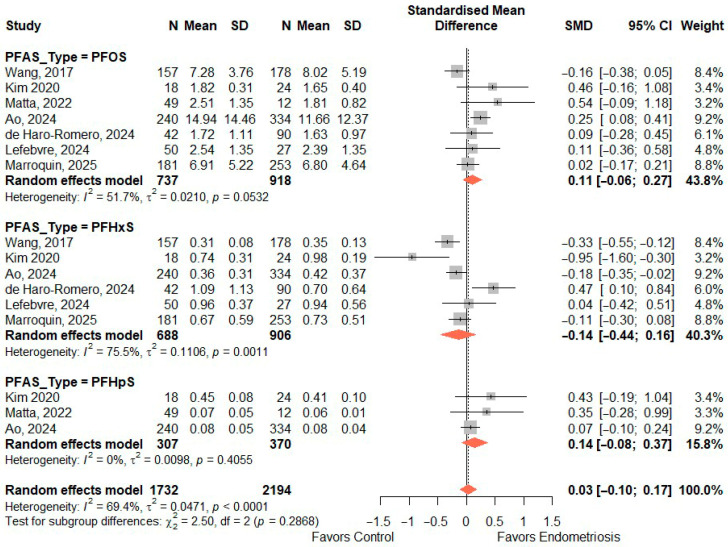
Standardized mean difference (SMD) in perfluoroalkyl sulfonic acids between endometriosis and controls. PFOS—perfluorooctanesulfonic acid [10,17,31,32,33,34,35], PFHxS—perfluorohexanesulfonic acid [10,17,31,32,34,35], PFHpS—perfluoroheptanesulfonic acid [17,32,33].

Figure 4 shows the pooled standardized mean differences (SMD) for sulfonic acid PFAS (PFOS, PFHxS, and PFHpS) comparing exposure levels between women with endometriosis and controls. Overall, there was no significant difference in sulfonic acid PFAS concentrations between endometriosis cases and controls (pooled SMD = 0.03, 95% CI: −0.10 to 0.17), although heterogeneity was substantial (I^2^ = 69.4%). Subgroup analyses showed no significant association for PFOS (SMD = 0.11, 95% CI: −0.06 to 0.27) or PFHpS (SMD = 0.14, 95% CI: −0.08 to 0.37). PFHxS showed a negative but non-significant pooled estimate (SMD = −0.14, 95% CI: −0.44 to 0.16) with high heterogeneity (I^2^ = 75.5%). The test for subgroup differences was not statistically significant (*p* = 0.2868), indicating no meaningful differences between individual sulfonic acid PFAS in relation to endometriosis when analyzed using continuous exposure levels.

### 3.8. SMD of Perfluoroalkyl Carboxylic Acids

**Figure 5:**
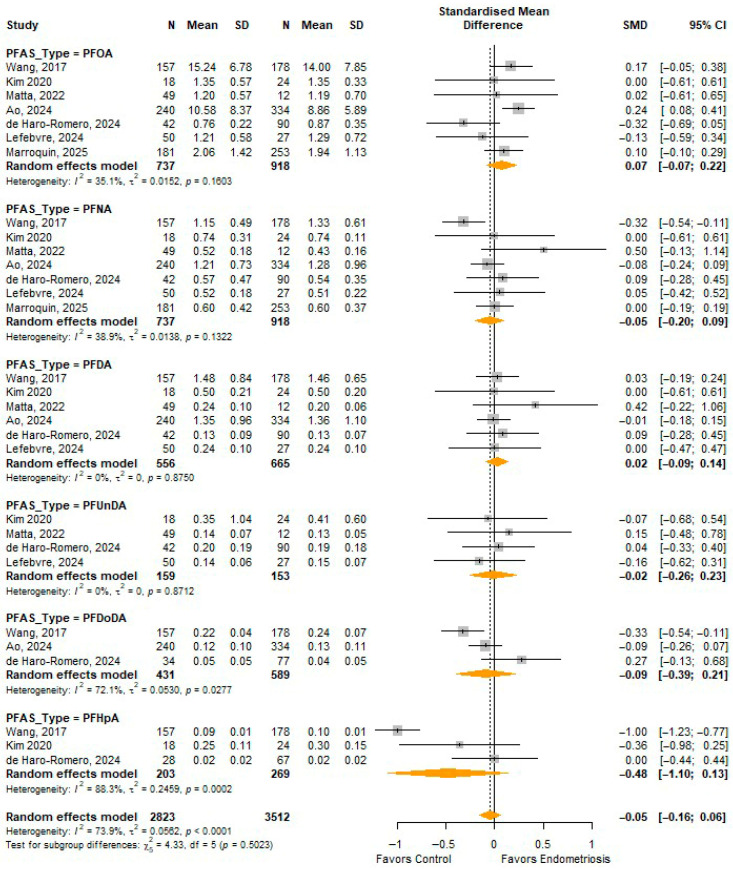
SMD in perfluoroalkyl carboxylic acids between endometriosis and controls. PFOA—Perfluorooctanoic acid [10,17,31,32,33,34,35]; PFNA—Perfluorononanoic acid [10,17,31,32,33,34,35]; PFDA—Perfluorodecanoic acid [10,17,31,32,33,34]; PFUnDA—Perfluoroundecanoic acid [10,32,33,34]; PFDoDA—Perfluorododecanoic acid [10,17,31]; PFHpA—Perfluoroheptanoic acid [10,31,32].

Figure 5 presents the pooled SMD for perfluoroalkyl carboxylic acids (PFOA, PFNA, PFDA, PFUnDA, PFDoDA, and PFHpA) comparing exposure levels between women with endometriosis and controls. Overall, there was no statistically significant difference in carboxylic acid PFAS concentrations between endometriosis cases and controls (pooled SMD = −0.05, 95% CI: −0.16 to 0.06), although heterogeneity was substantial (I^2^ = 73.9%). Subgroup analyses showed no significant associations for PFOA, PFNA, PFDA, PFUnDA, or PFDoDA. PFHpA showed a negative pooled estimate, but this was not statistically significant and was associated with high heterogeneity. The test for subgroup differences was not statistically significant (*p* = 0.5023), indicating no meaningful differences between individual carboxylic acid PFAS when analyzed using continuous exposure levels.

### 3.9. Sensitivity Analysis

The leave-one-out analyses demonstrated that the pooled estimates remained stable and statistically significant across sequential omission of individual studies, indicating that no single study disproportionately influenced the overall results. Subgroup sensitivity analyses by PFAS type showed similar stability within individual PFAS groups, with only minor changes in effect size and heterogeneity. Influence diagnostics, including studentized residuals, Cook’s distance, DFFITS, covariance ratios, and Baujat plots, identified a small number of studies contributing more to heterogeneity; however, removal of these studies did not materially change the overall pooled estimates. Overall, these findings indicate that the meta-analysis results are robust, and the observed associations between PFAS exposure and endometriosis are not driven by any single study or subgroup.

**Figure 6:**
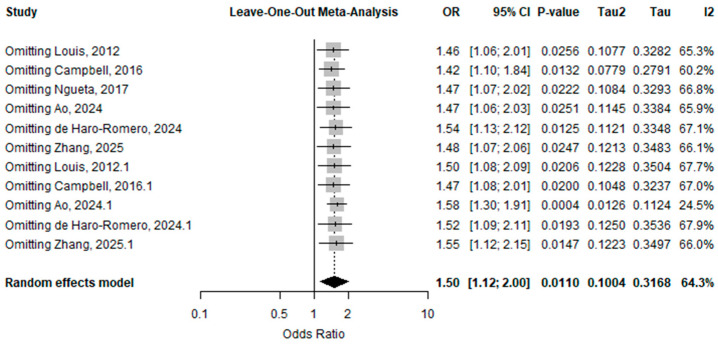
Leave-one-out sensitivity analysis for OR of PFSAs [10,16,17,28,29,30].

Leave-one-out sensitivity analysis showed that removing any single study did not substantially change the pooled effect, with odds ratios ranging from 1.42 to 1.58, all remaining statistically significant. The overall pooled estimate was OR = 1.50 (95% CI: 1.12–2.00), with moderate heterogeneity (I^2^ = 64.3%). These findings, shown in Figure 6, indicate that the association between PFOS exposure and endometriosis is robust and not driven by any single study.

**Figure 7:**
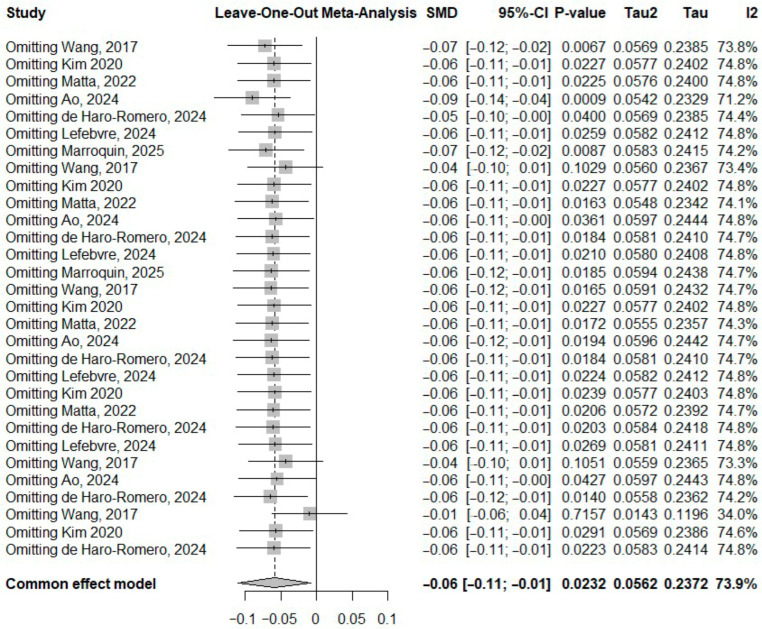
Leave-one-out sensitivity analysis for SMD of PFCAs [10,16,17,27,28,29,30,31,32,33,34,35].

Leave-one-out sensitivity analysis for PFCAs showed that the pooled estimates remained stable after sequential removal of individual studies, indicating that no single study substantially influenced the overall results. Influence diagnostics and the Baujat plot identified one study (Wang et al., for PFHpA) [31] contributing more strongly to heterogeneity; however, exclusion of this study did not materially change the pooled effect size, suggesting that the findings were robust. The plot is shown in Figure 7.

**Figure 8:**
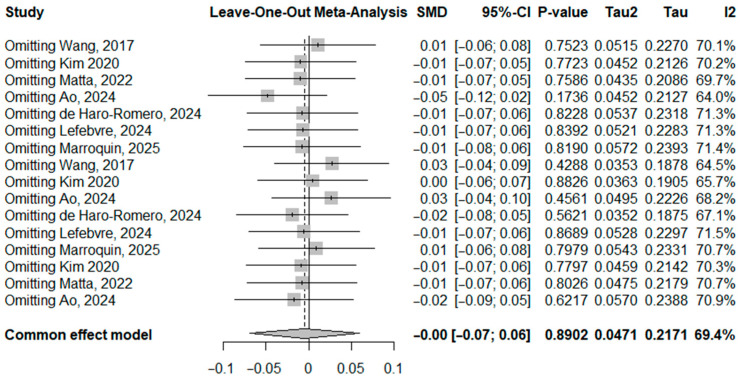
Leave-one-out sensitivity analysis for SMD of PFSAs [10,17,31,32,33,34,35].

Figure 8 presents the leave-one-out sensitivity analysis assessing the influence of individual studies on the pooled standardized mean difference. Sequential omission of each study did not materially change the overall pooled effect estimate, with pooled SMD values remaining close to zero and all confidence intervals crossing the null. The overall pooled estimate from the common effects model was SMD = −0.00 (95% CI: −0.07 to 0.06), with moderate heterogeneity (I^2^ = 69.4%). The *p*-values remained non-significant regardless of which study was removed, indicating that no single study had a disproportionate influence on the overall results. These findings suggest that the results of the meta-analysis are robust and not driven by any individual study.

### 3.10. Publication Bias

**Figure 9:**
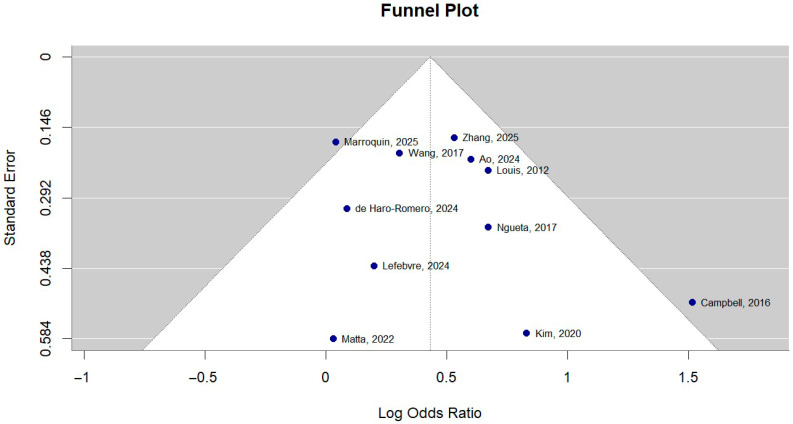
Funnel plot for publication bias [10,16,17,28,29,30,31,32,33,34,35].

Inspection of the funnel plot, shown in Figure 9, reveals a relatively symmetrical distribution of studies around the pooled effect estimate. Egger’s regression test did not indicate significant funnel plot asymmetry (z = −0.0397, *p* = 0.9683), and Begg’s rank correlation test was also not statistically significant (Kendall’s tau = −0.0545, *p* = 0.8793). These findings suggest no significant evidence of publication bias among the included studies.

## 4. Discussion

Our findings from this systematic review and meta-analysis indicate that certain PFAS are associated with increased odds of endometriosis. Pooled subgroup analyses demonstrated a significant positive association for both PFSA and PFCA and endometriosis. However, no statistically significant associations were observed individually for PFHxS or PFNA. In contrast, pooled SMD analyses did not consistently demonstrate higher PFAS concentrations among women with endometriosis. The significant associations observed for PFOS and PFOA, but not for PFHxS or PFNA, may be explained by differences in exposure levels, biological activity, and toxicokinetics, as PFOS and PFOA are typically present at higher concentrations, have stronger endocrine-disrupting and inflammatory effects, and have been more consistently linked to reproductive and gynecologic outcomes in epidemiological studies, whereas evidence for PFHxS and PFNA remains more limited and heterogeneous [13]. Collectively, these findings suggest that the relationship between PFAS exposure and endometriosis may be more apparent when exposure is categorized into tertiles or quartiles, rather than assessed as continuous SMDs, potentially indicating threshold effects as well as additive or synergistic toxicity resulting from co-pollutant exposures [39,40].

The difference between significant odds ratios and non-significant mean differences likely reflects differences in exposure modeling, as categorical analyses capture risk at higher exposure levels and potential threshold effects, whereas continuous analyses compare average concentrations and may not detect associations driven by individuals in the highest exposure categories. Moreover, the studies that reported odds ratios were different from the studies that reported SMD. Differences between studies may also be explained by how PFAS exposure was modeled, as some studies evaluated individual PFAS compounds [28] while others evaluated combined PFAS exposure [17]. Several studies have reported that combined exposure to multiple PFAS compounds is more strongly associated with endometriosis risk than individual PFAS alone, likely due to additive or synergistic endocrine-disrupting, inflammatory, and oxidative stress effects [16,17]. This may help explain why studies evaluating PFAS mixtures often report stronger and more consistent associations than studies evaluating individual compounds.

The observed association between PFOS and PFOA and endometriosis is biologically plausible [41]. Both compounds are long-chain, legacy PFAS with prolonged biological half-lives and strong binding affinity to serum proteins, with demonstrated toxic effects including the disruption of ovarian steroidogenesis, interfere with estrogen and progesterone signaling pathways, and promote chronic inflammatory responses [42,43]. However, the sulfonic acid group in PFOS confers increased bioavailability, bioaccumulation, and persistence. This increased bioaccumulation and persistence result in a high exposure/elimination ratio and thus longer periods of endocrine disruption [44]. Additionally, studies have shown that sulfonates notably alter cytokine production, thereby negatively impacting immunomodulation and promoting the chronic inflammation and fibrosis consistent with endometriotic lesions [44,45].

PFAS may function as baseline toxic agents due to their amphiphilic structure, which enables interaction with cell membranes and membrane-associated proteins [46]. These interactions may alter membrane fluidity, interfere with lipid metabolism, and disrupt cellular signaling pathways involved in inflammation, immune regulation, and hormone signaling [46]. Such effects are biologically relevant to endometriosis, which is characterized by chronic inflammation, altered immune surveillance, and estrogen-dependent growth of ectopic endometrial tissue [41]. PFAS exposure has also been associated with increased oxidative stress, immune dysregulation, and altered estrogen receptor signaling, all of which may promote the implantation and persistence of ectopic endometrial lesions [42]. Recent molecular and computational evidence suggests that PFAS may impair protamine–DNA interactions and promote DNA damage through oxidative mechanisms, providing biological plausibility for the observed associations between PFAS exposure and reproductive disorders [43]. Additionally, new human biomonitoring data show that PFAS exposure is associated with increased oxidative stress, reduced antioxidant capacity, and increased DNA damage, supporting oxidative stress–mediated mechanisms that may contribute to endometriosis pathogenesis [44]. These mechanisms provide biological plausibility for the observed associations between PFAS exposure and endometriosis risk. This, however, increases confidence in interpreting high-dose effects, thereby strengthening evidence for the observed effects when results are categorized into tertiles and quartiles [40]. Notably, PFTrDA was the only compound that showed a consistent significant association with endometriosis; however, this finding was derived from a single study by de Haro-Romero et al., and requires replication in independent populations [10]. Endometriosis is an estrogen-dependent, inflammatory, and immune-mediated disease, and PFAS exposure has been shown to influence all three of these biological pathways, providing mechanistic plausibility for a potential association.

Discrepancies between odds-ratio–based analyses and mean-difference analyses were observed for PFOS and PFOA. While categorical exposure comparisons demonstrated significant associations, pooled mean differences did not show consistent elevations in concentration among cases. This pattern reflects the non-linear or non-monotonic dose–response relationships commonly observed with endocrine-disrupting chemicals [45]. It also suggests that PFAS-related risk may be concentrated among individuals with higher exposure levels rather than evenly distributed across the exposure range [40].

Subgroup analyses by chemical classification further underscore the importance of compound-specific interpretation. Perfluoroalkyl sulfonic acids appeared more consistently associated with endometriosis than several perfluoroalkyl carboxylic acids [16,17]. Sulfonates generally have longer half-lives and may exert stronger immunomodulatory effects compared with certain carboxylates, potentially contributing to differential risk profiles [47]. However, the limited number of studies available for some compounds restricts definitive conclusions regarding class-specific effects.

PFAS exposure has also been linked to broader reproductive health effects, including menstrual cycle irregularities, altered age at menarche, reduced fecundability and fertility (Wang 2023) [48], and polycystic ovary syndrome (PCOS) [10]. However, findings across epidemiological studies have been inconsistent, with some studies reporting positive associations while others have reported null findings [49]. Therefore, while there is growing evidence suggesting that PFAS may affect female reproductive health through endocrine and metabolic disruption, these associations remain under investigation and have not been consistently demonstrated across all populations. Consistent with this evidence, Di Nisio et al. demonstrated that PFOA directly interferes with progesterone activity in human endometrial cells by antagonizing progesterone-responsive genes involved in implantation and receptivity [42]. They further reported that exposed young women exhibited delayed menarche and a significantly higher prevalence of irregular menstrual cycles, reinforcing the biological plausibility that PFAS may disrupt hormonal regulation of female reproductive function [42].

Findings for PFHxS and PFNA were not as consistent and were characterized by substantial heterogeneity. Several factors may explain this variability. First, differences in exposure matrices such as serum, follicular fluid, and endometrial tissue may influence measured concentrations and comparability across studies. Second, differences in exposure sources across studies may also have contributed to heterogeneity, as PFAS exposure can occur through contaminated drinking water, diet, occupational settings, and consumer products; however, most studies measured serum PFAS concentrations, which reflect cumulative long-term exposure regardless of the specific exposure route. Third, residual confounding remains a concern. Parity [50,51], body mass index [52,53,54], and other reproductive factors [55] that are strongly associated with both PFAS levels and endometriosis were not uniformly controlled across studies. Finally, traditional single-PFAS exposure models do not assess the impact of multicollinearity well, potentially masking toxic effects. Additive and synergistic mixture effects may play a more critical role in disease etiology, as suggested by studies reporting significant associations using mixture modeling approaches [56,57].

Nonetheless, this study has several notable strengths. It was conducted according to PRISMA guidelines with a prospectively registered protocol. We included all available studies from diverse geographic regions and incorporated both odds ratio and SMD analyses. Subgroup analyses by chemical class and sensitivity analyses were performed to explore heterogeneity. And publication bias was unlikely, based on funnel plot symmetry and findings from Egger’s and Begg’s tests.

However, several limitations must be acknowledged. The geographic distribution of studies was uneven, with most epidemiological evidence originating from the United States and parts of Europe and Asia. Regions with known PFAS contamination, such as Northern Italy, have produced important mechanistic and biomonitoring studies [58]; however, epidemiological studies examining endometriosis risk in these populations remain limited, restricting the global generalizability of the findings. Most included studies were case–control and cross-sectional in design, which limits causal inference and raises the possibility of reverse causation and selection bias, particularly in studies recruiting participants from infertility clinics or surgical populations.

Exposure assessment was typically based on a single biological measurement, which may not accurately reflect long-term cumulative exposure, especially for PFAS compounds with long biological half-lives but variable exposure sources over time. Differences in laboratory methods, biological sample types (serum, plasma, follicular fluid, or tissue), and timing of sample collection relative to diagnosis or surgery may also have contributed to heterogeneity across studies. Additionally, diagnostic methods for endometriosis varied, with some studies using surgically confirmed cases while others relied on self-reported diagnosis, which may introduce outcome misclassification.

Another important limitation is the potential for residual confounding. Although most studies adjusted for key variables such as age and body mass index, adjustment for other important reproductive and environmental confounders, including parity, smoking status, oral contraceptive use, and socioeconomic factors, was inconsistent across studies. Furthermore, PFAS compounds are highly correlated with one another, making it difficult to attribute risk to individual analytes due to collinearity and potential mixture effects. Most studies evaluated PFAS individually rather than as mixtures, which may underestimate the combined biological effects of multiple PFAS exposures. The observed association between PFTrDA and endometriosis was based on a single study and therefore may not be generalizable, highlighting the need for further studies examining this compound.

Finally, emerging PFAS compounds and short-chain PFAS, which are increasingly used as replacements for legacy PFAS, remain understudied in the context of endometriosis and represent a critical area for future research.

From a public health perspective, these findings are important given the widespread and persistence of PFAS exposure [11]. Even modest increases in risk may have meaningful implications at the population level, particularly for women of reproductive age. Endometriosis imposes substantial morbidity and healthcare costs, and identification of modifiable environmental risk factors could inform prevention strategies and regulatory policies [59]. An additional consideration is that regulatory actions in several countries have led to restrictions or phase-outs of certain legacy PFAS compounds, particularly PFOS and PFOA, due to their persistence and potential health effects [60]. As a result, exposure patterns are changing, with increasing use of short-chain and replacement PFAS compounds [61]. While these alternatives are often considered less bioaccumulative, their long-term health effects remain insufficiently understood. Therefore, future epidemiological studies should evaluate not only legacy PFAS but also emerging and replacement PFAS compounds to better understand evolving exposure patterns and their potential reproductive health implications.

Future research should prioritize prospective cohort studies with pre-diagnostic exposure assessment to reduce reverse causation bias. Approaches that include mixture modeling may better account for correlated exposures. Further investigation into short-chain and emerging PFAS, as well as mechanistic studies examining inflammatory and hormonal biomarkers, will be essential to clarify causality and biological pathways.

## 5. Conclusions

This meta-analysis suggests that exposure to certain PFASs, particularly PFOS and PFOA, may be associated with increased odds of endometriosis, while evidence for other compounds remains inconsistent. Despite heterogeneity and residual confounding suggesting the need for additional research, these findings contribute to the growing body of evidence that these persistent environmental endocrine-disrupting chemicals may play a role in the etiology of endometriosis.

## Figures and Tables

**Table 2:** PFAS investigated in the study.

	Perfluoroalkyl Sulfonic Acids			Perfluoroalkyl Carboxylic Acids					
Study	PFOS	PFHxS	PFHpS	PFOA	PFNA	PFDA	PFUnDA	PFDoDA	PFHpA
Louis, 2012 [28]	★	★	-	★	★	★	★	★	★
Campbell, 2016 [29]	★	★	-	★	★	★	-	-	-
Ngueta, 2017 [30]	★	-	-	★	-	-	-	-	-
Wang, 2017 [31]	★	★	-	★	★	★	★	★	★
Kim, 2020 [32]	★	★	-	★	★	★	★	-	★
Hammarstrand, 2021 [27]	★	★	-	★	-	-	-	-	-
Matta, 2022 [33]	★	★	★	★	★	★	-	-	-
Ao, 2024 [17]	★	★	★	★	★	★	★	★	★
Lefebvre, 2024 [34]	★	★	-	★	★	★	-	-	-
Marroquin, 2025 [35]	★	★	-	★	★	★	★	★	★
De Haro-Romero, 2024 [10]	★	★	-	★	★	★	★	★	★
Zhang, 2025 [16]	★	★	-	★	★	-	-	-	-

★—investigated in the study; -—not investigated; PFOS—Perfluorooctanesulfonic acid; PFHxS—Perfluorohexanesulfonic acid; PFHpS—Perfluoroheptanesulfonic acid; PFOA—Perfluorooctanoic acid; PFNA—Perfluorononanoic acid; PFDA—Perfluorodecanoic acid; PFUnDA—Perfluoroundecanoic acid; PFDoDA—Perfluorododecanoic acid; PFHpA—Perfluoroheptanoic acid.

## Data Availability

Data can be obtained upon request. All data used can be found in the following databases: PubMed, Embase, EBSCO Host, and Google Scholar.

## Abbreviations

The following abbreviations are used in this manuscript:

PFASs	Per- and polyfluoroalkyl substances
PFSAs	Perfluoroalkyl Sulfonic Acids
PFCAs	Perfluoroalkyl Carboxylic Acids
PFOS	Perfluorooctanesulfonic Acid
PFOA	Perfluorooctanoic Acid
PFHxS	Perfluorohexanesulfonic Acid
PFNA	Perfluorononanoic Acid
PFDA	Perfluorodecanoic Acid
PFUnDA	Perfluoroundecanoic Acid
PFHpA	Perfluoroheptanoic Acid
PFDoDA	Perfluorododecanoic Acid
PFHpS	Perfluoroheptanesulfonic Acid
PRISMA	Preferred Reporting Items for Systematic Reviews and Meta-Analyses
NOS	Newcastle-Ottawa Scale
ICD	International Classification of Diseases
OR	Odds Ratio
SMD	Standardized Mean Difference
CI	Confidence Intervals
